# Assessment of eating habits, lifestyle and physical activity among medical and dental students of Faisalabad Medical University

**DOI:** 10.12669/pjms.40.3.7323

**Published:** 2024

**Authors:** Hafiz Muhammad Usama Zuhair, Kiran Fatima, Umer Hussain, Ayesha Ayub

**Affiliations:** 1 Hafiz Muhammad Usama Zuhair, 4^th^ Year MBBS Punjab Medical College, Faisalabad, Pakistan; 2 Kiran Fatima, 4^th^ Year MBBS Punjab Medical College, Faisalabad, Pakistan; 3 Umer Hussain, 4^th^ Year MBBS Punjab Medical College, Faisalabad, Pakistan; 4 Ayesha Ayub, (Demonstrator HPERD) HPERD Department, Faisalabad Medical University, Faisalabad, Pakistan

**Keywords:** Feeding Behavior, Life Style, Exercise, Students, Medical

## Abstract

**Objective:**

To assess the eating habits, lifestyle and physical activity and their impact on health of students of Faisalabad Medical University (FMU) Faisalabad Pakistan.

**Methods:**

A descriptive cross-sectional study was prepared on 328 medical and dental students at Faisalabad Medical University from June, 2022 to October, 2022. Three Factor Eating Questionnaire Revised 21 (TFEQ-R21) was used to assess the eating habits among medical students and physical activity was assessed using the International Physical Activity Questionnaire (IPAQ) short version. A self-constructed questionnaire was used to assess lifestyle patterns.

**Results:**

According to the study, 191(58.2%) students out of 328 practiced unrestricted eating. While 229(69.8%) students demonstrated cognitive restraint and 128(39%) students indulged in emotional eating. Less than half of the students, 52(15.9%) lead unhealthy lifestyles compared to 276(84.1%) who had healthy habits. Out of 328 students, 104 (31.7%) engaged in low, 137(41.8%) in moderate, and 87(26.5%) in high levels of physical activity. Whereas a higher proportion of female students engaged in low to moderate physical activity (61.5% and 52.6%, respectively), while a higher proportion of male students (63.2%) were involved in intense physical activity.

**Conclusions:**

A large proportion of medical students exhibited poor dietary habits with low physical activity. Obesity and other metabolic diseases are more likely to strike medical students who engage in these unhealthy eating habits. As future health providers, health interventions must be implemented to avert future harm.

## INTRODUCTION

The demands of medical college may unintentionally impact a student’s health and way of life. Students must maintain a balanced schedule and adopt healthy lifestyles in order to thrive both cognitively and physically.^1^ Understanding nutrition becomes increasingly important as people transition from high school to college because food sources change and dietary difficulties arise. The university dining halls are where conventional on-campus students have their meals. Students can pick from a variety of nutritious and unhealthy lunch alternatives.^2^

The method by which someone obtains nutrients from food depends on their eating habits. Dietary habits can have a significant impact on health. In addition to obesity, cardiovascular disease, and diabetes mellitus, poor eating habits can lead to nutritional deficiencies.^3^ Lack of time makes it more challenging for young students to choose healthy diets and raises their risk of encountering challenging circumstances in the future. The opposite is expected, especially from medical students who have a significant influence on the general populace.^4^

Getting enough exercise, controlling stress, and overall maintaining a healthy lifestyle are additional parameters that influence someone’s wellbeing in addition to their dietary habits Each of these components must be considered when fostering social health.^5^ Both nutrition and inactivity have a significant impact on the emergence of obesity which is a notorious risk factor for cardiovascular events and a strong component of metabolic syndrome.^6^ A number of intervention studies have investigated the effects of increased aerobic exercise or decreased sedentary habits as part of multi-factorial therapy for the prevention of weight gain or remission of overweight. According to the WHO, inactivity is one of the top 10 global causes of death. You must exercise moderately for at least 30 minutes each day in order to live a healthy life.^7^

This study was intended to evaluate the understanding and application of healthy food, exercise, and lifestyle choices by medical students, since college is the best time to establish dependable health practices. The results will help in determining the primary factors that must be addressed in order to achieve a healthy lifestyle for medical students as they have a direct or indirect effect on sizable population.

## METHODS

A descriptive cross-sectional study was conducted on 328 students of MBBS and BDS programs at Faisalabad Medical University from June 2022 to October 2022. Simple random sampling was used and Cochran’s formula was used to determine sample size. Keeping the confidence interval at 95%, it was calculated as 322.

### Ethical Approval:

Study was approved by Ethical Review Committee of Faisalabad Medical University (No.F.48-ERC/FMU/2021-22/239 Dated 19-07-2022).

The short version of the International Physical Activity Questionnaire (IPAQ) was used to assess physical activity. The International Physical Activity Questionnaire (IPAQ) was basically developed into short and long versions.^8^ For the convenience of students, we used the short version in this study which consisted of seven questions covering all aspects of physical activity. Physical activity was measured using Metabolic Equivalent (MET).^8^ The working metabolic rate of an individual is calculated in relation to their resting metabolic rate or MET. MET is defined as the energy expended when doing nothing, which is equal to 1 kcal/kg/hour of caloric consumption. 3.3 METs were given for walking, four for moderate activity, and eight for intense activity.^9^

The Three Factor Eating Questionnaire Revised 21 (TFEQ-R21) was used to assess the eating habits of medical students. It was changed from the longer original version which consisted of 51 questions to a shorter and revised version that consisted of 18 questions (TFEQ-R18).^10^ Although the TFEQ-R18 scales were developed for obese individuals, factor evaluation of the TFEQ-R21 in adult population shows that it is also applicable to non-obese individuals and has also been validated in the general population. The questionnaire referred to current dietary patterns and assessed three components of eating behavior: restrained eating (a conscious dietary restriction used to control or encourage weight loss), uncontrolled eating (inability to control eating), and emotional eating (inability to deal with emotional stimuli) while using a four-point scale(certainly true/mostly true/mostly false/obviously false) .Uncontrolled eating (UE) had nine questions, emotional eating (EE) had six, and conscious restriction (CR) also had six questions making a total of 21 items.^11^

A self-structured questionnaire was used to assess students’ lifestyles, which measured sleep adequacy, weight concerns, information about medical conditions, drug use, and any psychological factors, such as stress or depression that could have affected quality of life in some way. The questionnaire was scored similarly to the TFEQ, with answers ranging from 1 to 5 (Never do = 5,

Rarely do = 4, Sometimes do = 3, Often do = 2, Very often do = 1). Students who scored more than half of the maximum were thought to have an active or healthy lifestyle.

### Statistical Analysis:

SPSS version 26 was used to analyse the data. Simple descriptive analysis for frequencies was used to calculate the percentages of TFEQ and IPAQ. Using the chi-square test, the results were compared to the nominal variable of gender. The percentages and frequencies obtained were organized in separate custom tables for each category.

## RESULTS

Total number of participants were 328 in which 168(51.2%) were females and 160 (48.8%) were males. Ages of medical students ranged from 17 to 25 years. Eating assessment revealed that 191 (58.2%) students practiced uncontrolled eating given 88 (46.1%) females and 103 (53.9%) males respectively. While 229 (69.8%) students practiced cognitive restraint. Out of which 118 (51.5%) were females and 111 (48.5%) were males. In total 128(39%) students were involved in emotional eating with both males and females having equal percentage that is 50%. Chi square revealed that for uncontrolled eating difference between male and female students was significant (p value = 0.028) whereas difference for cognitive restraint (p value = 0.865) and emotional eating (p value = 0.724) was not significant.

While in lifestyle assessment out of total 328 students less than half 52(15.9%) students with 31 (59.6%) females and 21 (40.4%) males were living an unhealthy lifestyle. While majority 276(84.1%) students with 137(49.6%) females and 139(50.4%) males were practicing healthy lifestyle. For the difference in lifestyle between men and women, Chi square yielded a non-significant p-value of 0.187.

Physical activity evaluation revealed that 104 (31.7%) out of total 328 students had low level activity, 137 (41.8%) had moderate physical activity and 87 (26.5%) had high level of physical activity. Percentage of females having low and moderate level of activity was greater than male students. While number of females having high activity was lesser than the males having high activity. Chi square test revealed significant difference in physical activity between males and females (P value = 0.003).

**Table-I:** Distribution of participants according to eating behavior based on TFEQ-R21 (n=328).

Eating behavior	Female	Male	Total	p-value
Uncontrolled Eater	88(46.1%)	103(53.9%)	191(100.0%)	0.028
Cognitive restraint Eater	118(51.5%)	111(48.5%)	229(100.0%)	0.865
Emotional Eater	64(50.0%)	64(50.0%)	128(100.0%)	0.724

## DISCUSSION

To our knowledge, this is the first study that applied shortened and refined version of one of the most widely used eating behavior measurement model (TFEQ-R21) and physical activity model (IPAQ) on medical students in Pakistan. When young students start their academic journey, their dietary and behavioral patterns may change. Freshmen in college typically gain weight throughout their first year. This increase is frequently attributed to irregular eating habits, which can be driven by stress, a change in lifestyle, and changes to the usual diet and eating routine.^12^ For eating habits, we classified the students as emotional eaters, cognitively restrained eaters, and uncontrollable eaters. 191 out of 328 people, according to the study, participated in uncontrolled eating. Compared to 229 persons who practiced cognitive restraint, 128 people had habit of emotional eating. According to a Taiwanese study, Men are more likely to participate in emotional and binge eating. However, a higher percentage of women practiced restricted eating.

These findings are similar to the findings of our study, which showed that more women than men restrict their food intake. This eating behavior is heavily influenced by the intense desire for an ideal body type. According to this research, more men indulge in impulsive eating.^13^ Our study shows more males participate in disordered eating but more women utilize cognitive restraint. Both had roughly comparable levels of emotional eating. The findings of another study done in University of Melbourne contradicted our theory that women overeat more frequently than men do because they are more likely to develop bulimia.^14^ Another study found that those with serious mental disorders were 49.22% more likely to experience emotional eating because the prevalence of obesity is higher in people with psychological disorders, and these people are at greater risk for emotional eating behavior. Obesity-related anxiety about physical appearance increases eating-related stress in those with severe mental disorders. 15

Our evaluation of students’ lifestyles revealed that more students than expected had active and healthy lives. The findings are consistent with another study conducted in Lahore, where the rates of healthy, reasonably healthy, and bad lifestyles were, respectively, 30.7%, 62.3%, and 7%. In our study, however, significant proportion of men (50.4%) led a healthy lifestyle, whereas the majority of women led an unhealthy one (59.6%). This gender-based assessment conflicts with the findings of a similar study conducted in Lahore, where the majority of males (11%) adopted unhealthy lifestyles while majority of females (33.5%) adopted healthy lifestyle. 16 Healthy lifestyle behaviors should be embraced earlier in order to prevent difficulties in the population, according to a study conducted on Brazilian students.^17^

Our study revealed that 104(31.7%) students had low physical activity, while 137(41.8%) students engaged in moderate activity, and just 87(26.5%) were highly active with a male student majority. The findings of our study are consistent with a study conducted in Polish adolescents where more males than females exhibited high physical activity levels^18^ and, a study conducted among students of medicine and public health where 48.6% of students engaged in moderate physical activity, while 32.6% engaged in intense physical activity, with high percentage of men engaging in vigorous physical activity.^19^ A cross- sectional study involving 1100 medical students from five medical colleges in Pakistan reported that medical students have an increased tendency towards sedentary lifestyle.^20^ Over half of university students in China, Italy, Nigeria, Germany, the United States of America, and twenty one other European nations didn’t engage in the recommended amount of physical activity, according to a study that analyzed their levels of physical activity.^21^

Another study in Sudanese medical students found that 44.9% of students had low activity levels, 32% had moderate activity levels, and 23.1% had high physical activity levels, which is quite comparable to our study.^5^ According to another study, just roughly 77.2% of Hungary’s medical workers get enough exercise. Men make up 55.2% of the inactive healthcare workers, while women make up 80.5%.^22^ This is consistent with our findings, which showed that women engage in less high-intensity physical activity than men. Similarly, a study conducted among Indian college students revealed that females faced more barriers to physical activity than males, resulting in low levels of physical activity.^23^

Our study showed that large proportion of medical students exhibited poor dietary habits with low physical activity. Medical students who practiced these unhealthy eating habits were at risk of acquiring obesity and other metabolic diseases. As future health providers health interventions must be implemented to avert future harm. Further, the current study’s findings demonstrated that both of these models were effective in their respective domains and can be used in clinical settings to assess eating habits and physical activity theoretically. This study was conducted in a single institution. In order to assess these trends among medical students, the authors recommend conducting a multi-institutional study.

### Limitations:

Firstly, its cross-sectional design precludes causal inferences. Secondly, the study is carried out in a single institute. It should be replicated in multiple colleges and cities to validate the findings. Thirdly, due to limited resources at undergraduate level numerous factors contributing to poor dietary patterns and physical lifestyle among medical students could not be identified. Further studies are required to investigate these causes for improving medical students’ health.

## CONCLUSION

According to TFEQ-R21 criteria 191(58.2%) students, or more, engaged in uncontrolled eating. While 229(69.8%) practiced cognitive restraint and 128(39%) students were involved in emotional eating. These figures were concerning. Medical students who followed these poor eating habits are more likely to develop obesity and other illnesses. Similarly, evaluation of physical activity according to IPAQ among students revealed that the majority engaged in very low to moderate levels of physical activity, with only a small minority engaging in high levels of activity that disproportionately involved male and female students. When lifestyles were compared, a higher percentage of female students had unhealthy lifestyle in comparison to their male colleagues. This type of conduct implied that necessary steps must be taken to enhance eating behaviors as well as lifestyle in general. Encouragement of physical activity among students is also crucial. Otherwise, these actions would eventually have a negative impact on medical students, who are themselves health pioneers.

### Authors Contribution:

**HMUZ:** Conceived, designed and editing of manuscript as well as responsible for integrity of research.

**KF:** Data collection and manuscript writing.

**UH:** Statistical analysis and result compilation.

**AA:** Review and final approval of manuscript.
